# Revision of the genus *Epiparbattia* Caradja, 1925 (Lepidoptera, Crambidae, Pyraustinae), based on morphology and molecular data

**DOI:** 10.3897/zookeys.960.54986

**Published:** 2020-08-17

**Authors:** Dandan Zhang, Kai Chen, Lanbin Xiang

**Affiliations:** 1 School of Ecology, Sun Yat-sen University, Guangzhou, Guangdong 510275, China Sun Yat-sen University Guangzhou China; 2 School of Life Sciences, Sun Yat-sen University, Guangzhou, Guangdong 510275, China Sun Yat-sen University Guangzhou China

**Keywords:** China, *
Epiparbattia
*, molecular phylogeny, new species, *
Sclerocona
*, taxonomy

## Abstract

The genus *Epiparbattia* Caradja, 1925 is revised based on general appearance, including genitalia. A new species, *Epiparbattia multispinalis* Zhang & Chen, **sp. nov.** is described. The external characters and genitalia morphology of all species are figured. The phylogeny of *Epiparbattia* species is investigated using molecular data. Monophyly of the genus is well supported by phylogenetic analysis based on sequence data of *COI*, *16S rRNA*, *EF-1*α and *28S rRNA* gene regions.

## Introduction

The genus *Epiparbattia* Caradja, 1925 comprises only two species, *E. gloriosalis* Caradja, 1925 and *E. oligotricha* Zhang & Li, 2005, distributed in southern China, India and Bhutan (Munroe and Mutuura 1971; Nuss et al. 2003–2020; Irungbam et al. 2016). These two species are easily recognized by the yellow body and the creamy white wings bearing dark brown markings. Three specimens, having a different colour but similar genitalic morphology, represent a new species of *Epiparbattia* which is described below. In this paper, we also redefine the genus and summarize the diagnoses of all species based on the external morphological and genitalic characters. The phylogeny of *Epiparbattia* based on sequence data of *COI*, *16S rRNA*, *EF-1*α and *28S rRNA* gene regions is reconstructed.

## Material and methods

### Morphological studies

The material studied, including the types of the newly described species, are all deposited at the Museum of Biology, Sun Yat-sen University, China (**SYSBM**) except those specified as being in the Insect Collection of the College of Life Sciences, Nankai University, China (**NKU**), the Institute of Zoology, Chinese Academy of Sciences (**IZCAS**) and the National History Museum, London, United Kingdom (**NHMUK**). Slides of genitalic dissections were prepared according to Robinson (1976) and Li and Zheng (1996), with some modifications. Genitalia terms follow Klots (1970), Munroe (1976), Maes (1995) and Kristensen (2003). Images of the specimens at different focal levels were made using a Canon EOS 1DX camera in combination with the Helicon Remote image stacking program; the genitalia pictures were taken using Zeiss Axio Scope.A1 in combination with a Zeiss AxioCam camera and the Axio Vision SE64 programme on a Windows PC; source images were then aligned and stacked on Helicon Focus to obtain a fully sharpened composite image.

### Molecular analyses

In total seven species of four genera were included for molecular phylogenetic analyses (Table 1). *Euclasta stoetzneri* (Caradja, 1927) was chosen as the outgroup because the genus *Euclasta* Lederer, 1855 is considered as the basal lineage of the Pyraustinae (Mally et al. 2019). One species of *Sclerocona* and two species of *Crocidophora* were included as closely related groups based on the fovea of the forewing, the minute basal and apical outer spurs of the hindleg in males and the similar genitalic characters. Total DNA was extracted from two legs and, sometimes in addition, from the abdomen of the dry specimens using the TIANGEN DNA extraction kit following the manufacturer’s instructions. The nucleotide sequences of two mitochondrial genes, cytochrome c oxidase subunit I (*COI*) and 16S ribosomal RNA (*16S rRNA*), and two nuclear genes, Elongation factor-1 alpha (*EF-1*α) and 28S ribosomal RNA (*28S rRNA*) were selected for study. Primers used in this study are as follow: LCO/Nancy for *COI* and LR-J-12888/ LR-N-13398 for *16S rRNA* (Simon et al. 2006), Oscar-6143/Bosie-6144 for *EF-1*α (Hundsdoerfer et al. 2009) and 28S-f1/28S-r1 for *28S rRNA* (Lee and Brown 2008). All PCRs were performed in 25 µl of solution, containing 2 µl 10×PCR Buffer, 2 µl dNTP (2.5 mM each), 0.6 µl MgCl_2_ (25 mM), 1 µl of each primer (10 pmol/µl), 0.2 µl Takara Taq DNA Polymerase (Takara Bio Inc., 5 u/µl), 4 µl of template DNA and 14.2 µl ddH_2_O for *COI*, *16S rRNA* and *28S rRNA*, and 10 µl 2×PCR Buffer (8 mM MgCl_2_), 2 µl dNTP (10 mM each), 1 µl of each primer (10 pmol/µl), 0.4 µl KOD FX DNA Polymerase (TOYOBO CO., LTD., 1 u/µl), 4 µl of template DNA and 6.6 µl ddH_2_O for *EF-1*α. PCR cycle conditions were set to an initial denaturation of 5 min at 95 °C, 35 cycles of 30 seconds at 94 °C, 30 seconds at 48 °C (*COI* and *16S rRNA*) or 52 °C (*EF-1*α and *28S rRNA*) and 1 min at 72 °C for amplification, and a final extension at 72° C for 10 min. PCR products were confirmed with 1.5% agarose gel electrophoresis in TAE buffer, then were purified and direct-sequenced at Majorbio Bio-pharm Technology Co., Ltd (Guangzhou), utilizing the same primers used for PCR amplification.

**Table 1.** Species sampled for the molecular phylogenetic analysis.

Genus	Species	Voucher no.	Locality	Genbank accession number				References
				*COI*	*16S*	*EF-1*α	*28S*	
* Euclasta *	* stoetzneri *	SYSU-LEP0334	Shaanxi	MT738696	MT734412	MT724335	MT734404	Present study
* Crocidophora *	* lutusalis *	SYSU-LEP0088	Yunnan	MT738697	MT734413	MT724336	MT734405	
	*pallidulalis*	SYSU-LEP0090	Yunnan	MT738698	MT734414	MT724337	MT734406	
* Epiparbattia *	* oligotricha *	SYSU-LEP0243	Sichuan	MT738699	MT734415	N/A	MT734407	
	* oligotricha *	SYSU-LEP0359	Yunnan	MT738700	MT734416	MT724338	MT734408	
	* multispinalis *	SYSU-LEP0351	Hubei	MT738701	MT734417	N/A	MT734409	
	* multispinalis *	SYSU-LEP0378	Hubei	MT738702	MT734418	MT724339	MT734410	
	* gloriosalis *	SYSU-LEP0244	Guangdong	MT738703	MT734419	MT724340	MT734411	
* Sclerocona *	* acutella *	SYSU-LEP0152	Macao	MG739577	MG739589	MG739601	MG739612	Chen et al. 2018

The sequences were aligned using Clustal W (Thompson et al. 1994) in MEGA 6 (Tamura et al. 2013) with default settings. The aligned matrix was corrected by eye. Gaps were treated as missing data. Phylogenetic analyses were inferred using Bayesian inference (BI) method in MrBayes 3.2.6 (Ronquist et al. 2012) and maximum likelihood (ML) in RAxML 8.2.10 (Stamatakis 2014). BI analysis was run with independent parameters for the *COI* and the *16S rRNA* gene partitions under the GTR + G model, the *EF-1*α gene partition under the GTR + I model, and *28S rRNA* gene partition under the GTR + G model, as suggested by jModelTest 0.1.1 (Posada 2008). Two independent runs, each with four Markov Chain Monte Carlo (MCMC) simulations, were performed for 20 million generations sampled every 1000^th^ generation. The first 25% trees were discarded as burn-in, and posterior probabilities (PP) were determined from remaining trees. ML analysis was executed under the GTR + G model for all gene partitions and with 1000 iterations for the bootstrap test. The pairwise Kimura 2-Parameter (K2P) distances between species were calculated from the *COI* gene using MEGA 6 (Tamura et al. 2013).

## Results

### Epiparbattia

Taxon classificationAnimaliaLepidopteraCrambidae

Caradja, 1925

775D86A3-DB16-5891-AE71-1CDF1E48657C

Epiparbattia Caradja, 1925: 358.

#### Type species.

*Epiparbattia gloriosalis* Caradja, 1925, by monotypy.

**Diagnosis.** This genus is related to *Sclerocona* Meyrick, 1890 in the forewings bearing a fovea basally beyond the cell and another fovea between R_3+4_ and R_5_, as well as the minute basal and apical outer spurs of the hindlegs in the male, the developed and sclerotized lamella postvaginalis, the coiled and partly sclerotized posterior part of the ductus bursae and the second signum with two almost parallel ridges in the female genitalia, but can be distinguished by the larger body size, the relatively short labial palpi and the prominent markings on the wings. In the genitalia, *Epiparbattia* differs from *Sclerocona* by the relatively broad uncus, the uninflated sacculus, the stout, ventrodistally sclerotized phallus and the broad, nearly elliptical signum.

**Redescription.** Frons rounded, sometimes weakly flat. Labial palpi obliquely porrect, third segment porrect; exceeding frons by less than the diameter of eyes. Maxillary palpi slightly broadened terminally. Hindlegs of male with basal and apical outer spurs minute. Forewings elongated triangular, costa slightly curved near apex, apex obtuse, termen slightly curved and oblique, tornus rounded; reniform stigma developed and connected with postmedial band, postmedial band comprising of a series of patches and with a broad patch on posterior margin, subterminal band comprising of a series of broad, elliptical patches; length of cell less than half of forewing, male with posterior margin of cell, CuA_2_ and 1A basally curved, and forming a fovea, with a scale-tuft on the underside surface at position of fovea, R_3_ and R_4_ stalked about 1/2–2/3 length of R_4_, R_5_ basally curved, a fovea present between R_3+4_ and R_5_ but without scale-tuft on the underside surface at position of fovea. Hindwings fan-shaped; length of cell less than half length of hindwing; Sc+R_1_ and Rs anastomosed for 1/3–1/2 length of Rs; subterminal band narrow. **Male genitalia.** Uncus nearly triangular, distally narrowly rounded, with distal half densely setose laterally and dorsally, basal half sparsely setose laterally. Tegumen with dorsal 1/3–1/2 narrow and basal 2/3–1/2 broad. Transtilla connected, arms nearly triangular or trapezoidal, sparsely bearing slender setae, with ventral process extending to juxta. Valvae tongue-shaped, costa nearly straight, ventral margin convex, apex rounded; sella weakly sclerotized and setose, dorsally bearing a large cluster of curved and thick setae or several slender setae forming an editum, ventrally with strongly sclerotized processes; sacculus not inflated; saccus nearly triangular. Juxta with dorsal part divided into two arms. Phallus stoutly cylindrical, distally with ventral part sclerotized. **Female genitalia.** Ovipositor lobes flat, densely setose. Apophyses stout, anterior apophyses about same length of posterior apophyses, 8^th^ tergite with base of anterior apophysis strongly extended forward and connected with lamella postvaginalis. Lamella postvaginalis developed, sclerotized. Antrum reduced. Ductus bursae usually longitudinally wrinkled, about 2–3 times length of corpus bursae; with most of posterior part coiled and partially sclerotized, usually inflated; colliculum elongated hourglass-shaped; ductus seminalis arising closely from anterior end of colliculum. Corpus bursae globular or oval, wrinkled; appendix bursae originating from posterior part; signum broadly rhomboid, nearly elliptical; second signum located between base of appendix bursae and entrance of ductus bursae, plate-shaped and curved, usually with two almost parallel ridges.

**Distribution.** China, India, Bhutan.

### Key to species of *Epiparbattia*

**Table:** 

1	Forewings ground colour pale yellow and the covering dark brown scales forming markings in male (Fig. 3), wings pale yellow in female (Fig. 4); ventral processes of sella with rows of densely set spines ventrally, dorsalmost process long and straight (Fig. 7)	***E. multispinalis* Zhang & Chen, sp. nov.**
–	Wings creamy white bearing dark brown markings; ventral processes of sella with the dorsalmost one curved and with sparse spines ventrally	**2**
2	Forewings with postmedial band interrupted (Fig. 1); ventral margin and costa of valva approximately parallel, dorsal part of sella densely covered with thick setae forming editum, the setae with apex curved and divided into several filaments, ventral part of sella with dorsalmost process slightly curved and extending inward (Fig. 5)	***E. gloriosalis* Caradja, 1925**
–	Postmedial band of forewing not interrupted (Fig. 2); valvae gradually widened from base to apex, dorsal part of sella sparsely covered with slender and simple setae forming editum, ventral processes of sella with the dorsalmost one extending dorsad (Fig. 6)	***E. oligotricha* Zhang & Li, 2005**

### Epiparbattia gloriosalis

Taxon classificationAnimaliaLepidopteraCrambidae

Caradja, 1925

BE053098-F825-56AD-8FC1-CDDE2E096B3E

Figs 1 , 5 , 8

Epiparbattia gloriosalis Caradja, 1925: 359.
Epiparbattia gloriosalis whalleyi Munroe & Mutuura, 1971: 506.

#### Type material examined.

***Paratype*** of *Epiparbattia gloriosalis* Caradja, 1925: 1♀, [China: Guangdong]: Lienping [Lianping], 26. April (NHMUK). Types of *Epiparbattia gloriosalis whalleyi* Munroe & Mutuura, 1971: ***holotype*** ♂, [India]: Assam, 5000 ft, Shillong, 19.May.1924, Fletcher coll., Pyralidae Brit. Mus. Slide no. 8708 (NHMUK); ***allotype*** ♀, [India]: Assam, 5000 ft, Shillong, at light, 18.V.[19]28, T. Bainbridge Fletcher, Pyralidae Brit. Mus. Slide no. 8709 (NHMUK); ***paratypes***: 2♀♀, [India]: Assam, Shillong, at light, H. M. Parish., Pyralidae Brit. Mus. Slide no. 5384 (NHMUK).

**Figures 1–4.**
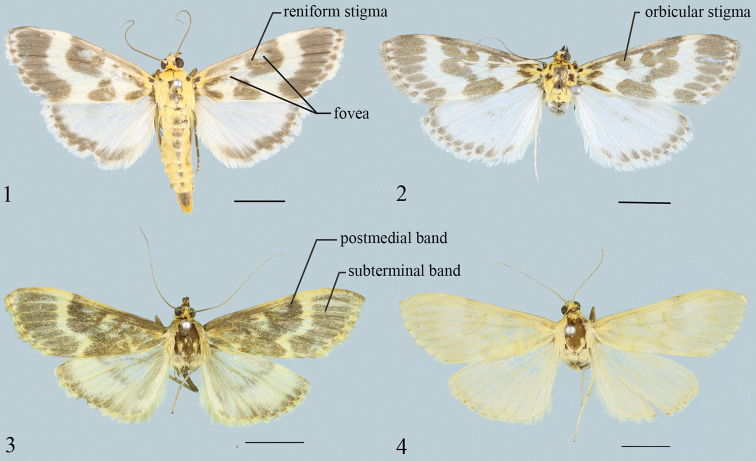
Adults of *Epiparbattia* spp. **1***E. gloriosalis*, male (Renhua, Guangdong) **2***E. oligotricha*, female (Tianquan, Sichuan) **3***E. multispinalis* sp. nov., holotype, male (Shuangping, Zhuxi, Hubei) **4***E. multispinalis* sp. nov., paratype, female (Shuangping, Zhuxi, Hubei). Scale bars: 5.0 mm.

#### Other material examined.

China: Fujian: 1♂, Sangang, 15.VIII.1979 (IZCAS); Guangdong: 2♂♂, Mt. Danxiashan, Renhua, alt. 408 m, 15. IV.2008, leg. Wang Fengwei; 1♂, 1♀, Mt. Dinghushan, Zhaoqing, 23.17°N, 112.55°E, alt. 56 m, 8.IV.2013, leg. Li Jinwei, genitalia slide no. CXH12039 (♂), SYSU1036 (♀, molecular voucher no. SYSU-LEP0244); Guangxi: 1♀, Mt. Shiwandashan, 21.91°N, 107.91°E, alt. 352 m, 18.IV.2012, leg. Li Jinwei; Yunnan: 1♂, Lufeng, 22.VI.1982, leg. Song Shimei (IZCAS); 1♀, Kunming, 10.V.1980, leg. Zhong Tiesen (IZCAS); 1♀, Muding, V.1975 (IZCAS); Xizang: 1♀, Pailong, Linzhi, 30.01°N, 95.00°E, alt. 2010 m, 5.VII.2013, leg. Li Jinwei.

#### Diagnosis.

Wingspan 32.0–41.0 mm. This species is similar to *Epiparbattia oligotricha* in appearance, but can be distinguished from it by the tegulae with only one black spot at base, the interrupted postmedial band and the narrower patch on the posterior margin; in male genitalia by the dorsal side of the sella densely covered with thick setae forming editum, and the setae subapically curved and divided into several filaments, the ventral processes of the sella with the dorsalmost curved inward, apically bifurcated, the ventralmost curved ventrad, and by the longer arms of the juxta; in female genitalia by the absence of a U-shaped concave unsclerotized window of the lamella postvaginalis anteriorly and the uninflated posterior part of the ductus bursae.

#### Distribution.

China (Fujian, Hubei, Guangdong, Guangxi, Sichuan, Yunnan, Xizang), India, Bhutan.

#### Biology.

Larvae bore in the stems of bamboo shoots of *Sinocalamus affinis* (Rendle) McClure (Wang 1980).

### Epiparbattia oligotricha

Taxon classificationAnimaliaLepidopteraCrambidae

Zhang & Li, 2005

31DEC0A4-1602-5A9E-A66C-7AF7D4B4D5C5

Figs 2 , 6 , 9

Epiparbattia oligotricha Zhang & Li, 2005: 40.

#### Type material examined.

***Holotype*** ♂, China: Guizhou: Mt. Fanjingshan, 27°33'N, 108°24'E, alt. 1700 m, 1.VI.2002, leg. Wang Xinpu (NKU); ***Paratypes***: Yunnan: 2♂♂, 2♀♀, Jinping, 22°28'N, 103°7'E, alt. 1700 m, 9–13.V.1956, leg. Huang Keren (IZCAS).

#### Other material examined.

China: Sichuan: 5♀♀, Labahe, Tianquan, 30.09N, 102.52E, alt. 1860 m, 8.VII.2012, leg. Li Jinwei, genitalia slide no. SYSU1035, molecular voucher no. SYSU-LEP0243, SYSU-LEP0335; Yunnan: 1♂, Qinlangdang Reserve Station, Gaoligongshan Reserve, Nujiang, 27.69°N, 98.27°E, alt. 380 m, 30.V.2017, leg. Teng Kaijian et al., genitalia slide no. ZDD12109, molecular voucher no. SYSU-LEP0359 (NKU).

#### Diagnosis.

Wingspan 32.0–47.0 mm. This species is superficially similar to *Epiparbattia gloriosalis*, but can be distinguished from it by the tegulae bearing a second large black spot in the center, the large orbicular stigma, the uninterrupted postmedial band and the wider patch of the postmedial band at the posterior margin; in male genitalia by the dorsal side of the sella sparsely covered with slender simple setae forming editum, the ventral processes of the sella with the dorsalmost curved dorsad and with ventral margin sparsely bearing spines, the ventralmost slightly curved inward, as well as the shorter arms of the juxta; in female genitalia by the presence of a deep, U-shaped concave unsclerotized window of the lamella postvaginalis anteriorly and the inflated posterior part of the ductus bursae.

#### Distribution.

China (Sichuan, Guizhou, Yunnan).

**Figures 5–7.**
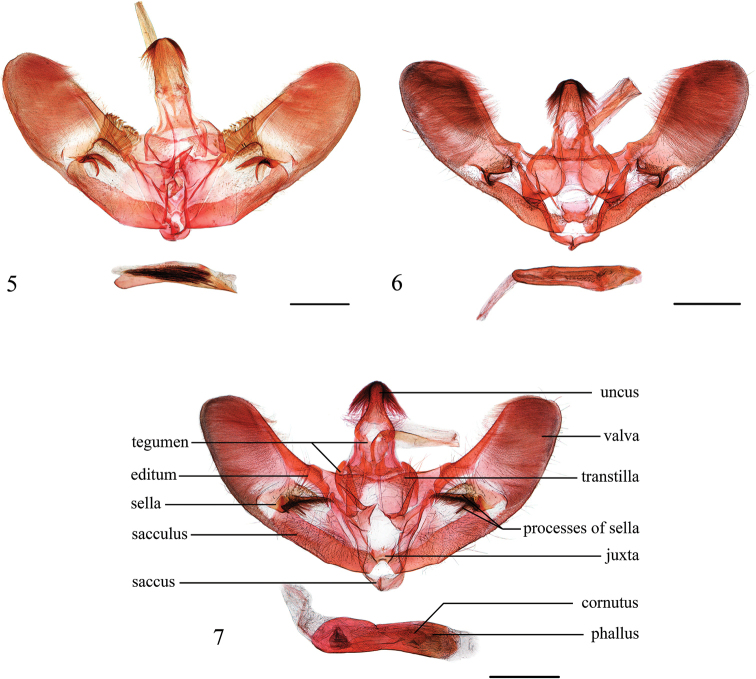
Male genitalia of *Epiparbattia* spp. **5***E. gloriosalis*, Guangdong (genitalia slide no. CXH12039) **6***E. oligotricha*, Yunnan (genitalia slide no. ZDD12109) **7***E. multispinalis* sp. nov., Hubei (genitalia slide no. ZDD12101). Scale bars: 1.0 mm.

### Epiparbattia multispinalis

Taxon classificationAnimaliaLepidopteraCrambidae

Zhang & Chen sp. nov.

37640FA7-0599-5FC5-9E87-A3CF6072A5A2

http://zoobank.org/CC9D9746-060F-43D4-A173-50F6AA904464

Figs 3 , 4 , 7 , 10

#### Material examined.

***Holotype*** ♂, China: Hubei: Shuangping, Zhuxi, 31.57N, 109.87E, alt. 1201 m, 5.VII.2017, leg. Qi Wanding, genitalia slide no. ZDD12101, molecular voucher no. SYSU-LEP0378 (NKU). ***Paratypes***: 2♀♀, same data as holotype, genitalia slide no. ZDD12074, ZDD12095, molecular voucher no. SYSU-LEP0351 (NKU, SYSBM).

#### Diagnosis.

The new species differs from the other two species by the pale yellow ground colour of forewings with dark brown markings in the male and the pale-yellow wings in the female. In the male genitalia, *Epiparbattia multispinalis* is similar to *E. oligotricha*, but differs from the latter in the concave lateral margin of the uncus, the more convex ventral margin of the valva, the ventral processes of the sella with the dorsalmost straight and long, narrowly triangular, with rows of spines ventrally, transversely extending inward, and by the large drop-shaped cornutus. In the female genitalia, this species is different from *E. gloriosalis* and *E. oligotricha* by the prominently inflated posterior part of the ductus bursae, approximately 2–3 times the width of the remainder of the ductus bursae.

#### Description.

Wingspan 29.0–33.0 mm. **Male** (Fig. 3). ***Head*.** Frons flat or round, brown. Vertex brown. Labial palpi brown, pale yellow at base beneath. Maxillary palpi brown, paler at apex. Basal scales of proboscis pale brown. Antennae yellowish brown. ***Thorax.*** Pale brown dorsally, tegulae bearing scales pale yellow with pale brown apex; greyish white ventrally. Legs yellowish brown, hindlegs of male with basal and apical outer spurs minute, about 1/5 length of inner spurs. Forewings ground colour pale yellow, with area from base to postmedial band densely covered with dark brown scales, only with a diffuse pale yellow medial area between wing base and postmedial band; reniform stigma dark brown, nearly triangular; postmedial band dark brown, from costal 2/3 to middle of posterior margin; subterminal band broad, brown, with veins pale yellow; termen with brown spots at veins end; fringe pale yellow, scattered with dark browns scales. Forewings with a fovea beyond posterior margin of cell and another outside of cell. Hindwings pale yellow, with basal half sparsely scattered with dark brown scales and a narrow pale brown postmedial line as outer demarcation; subterminal band dark brown, with inner margin suffusing and irregular; termen and fringe same as forewing. ***Abdomen.*** Brown, dorsally with posterior margin of each segment pale yellow. ***Male genitalia*** (Fig. 7). Uncus laterally strongly concave and with distal end narrowly rounded. Valvae slightly curved, costal margin slightly concave and ventral margin convex; width of basal half relatively even and slightly tapering from middle towards bluntly rounded apex; sella nearly rhombic and weakly sclerotized, dorsal part sparsely bearing slim setae and several thick setae forming editum, ventral processes of sella with the dorsalmost sclerotized, straight and long, narrowly triangular, transversely extending inward and densely bearing rows of spines ventrally, another process of sella spine-shaped and small. Juxta small, nearly inversely trapezoidal, dorsal arms weak and widely separated. Phallus slightly narrowed in middle, with a large drop-shaped, weakly sclerotized cornutus. **Female** (Fig. 4). Head and thorax yellow, antennae pale brown. Forewings yellow, with a pale yellow band indistinct, wider than that in male; termen with pale brown spots at veins end; fringe pale yellow. Hindwings pale yellow; subterminal band indistinct; termen and fringe as in forewing. Abdomen brown. ***Female genitalia*** (Fig. 10). Lamella postvaginalis densely covered with minute spines, strongly extended dorsad and connected dorsally, with dorsal part forming a pair of closely associated rounded sclerites. Ductus bursae about three times diameter of corpus bursae, with posteriormost part prominently inflated, about 2–3 times width of the remainder; colliculum hourglass-shaped. Corpus bursae globular, length of signum about 2/3 of diameter of corpus bursae, ends of the long axis approximately right-angled, ends of the short axis (perpendicular to long axis) produced into short acute-angled tips.

#### Etymology.

The specific name is derived from the Latin *multi*- (= many) and *spinalis* (= spine) corresponding to the ventral processes of the sella bearing many spines.

**Figures 8–10.**
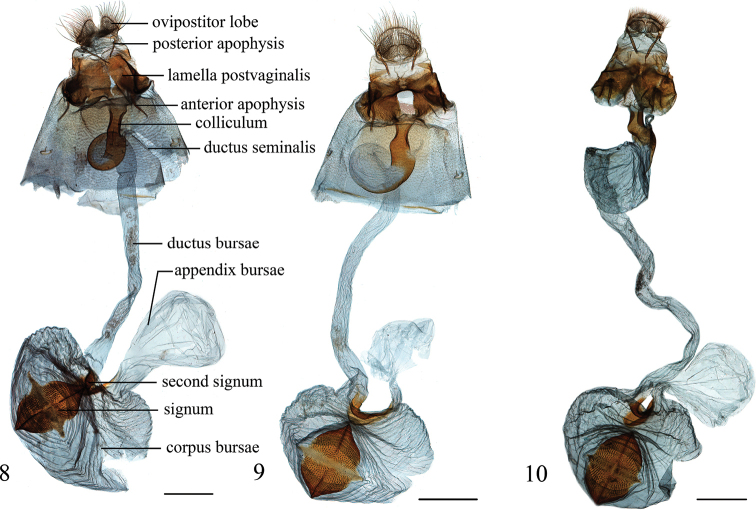
Female genitalia of *Epiparbattia* spp. **8***E. gloriosalis*, Guangdong (genitalia slide no. SYSU1036) **9***E. oligotricha*, Sichuan (genitalia slide no. SYSU1035) **10***E. multispinalis* sp. nov., Hubei (genitalia slide no. ZDD12074). Scale bars: 1.0 mm.

#### Distribution.

China (Hubei).

##### Phylogenetic relationships

**Figure 11.**
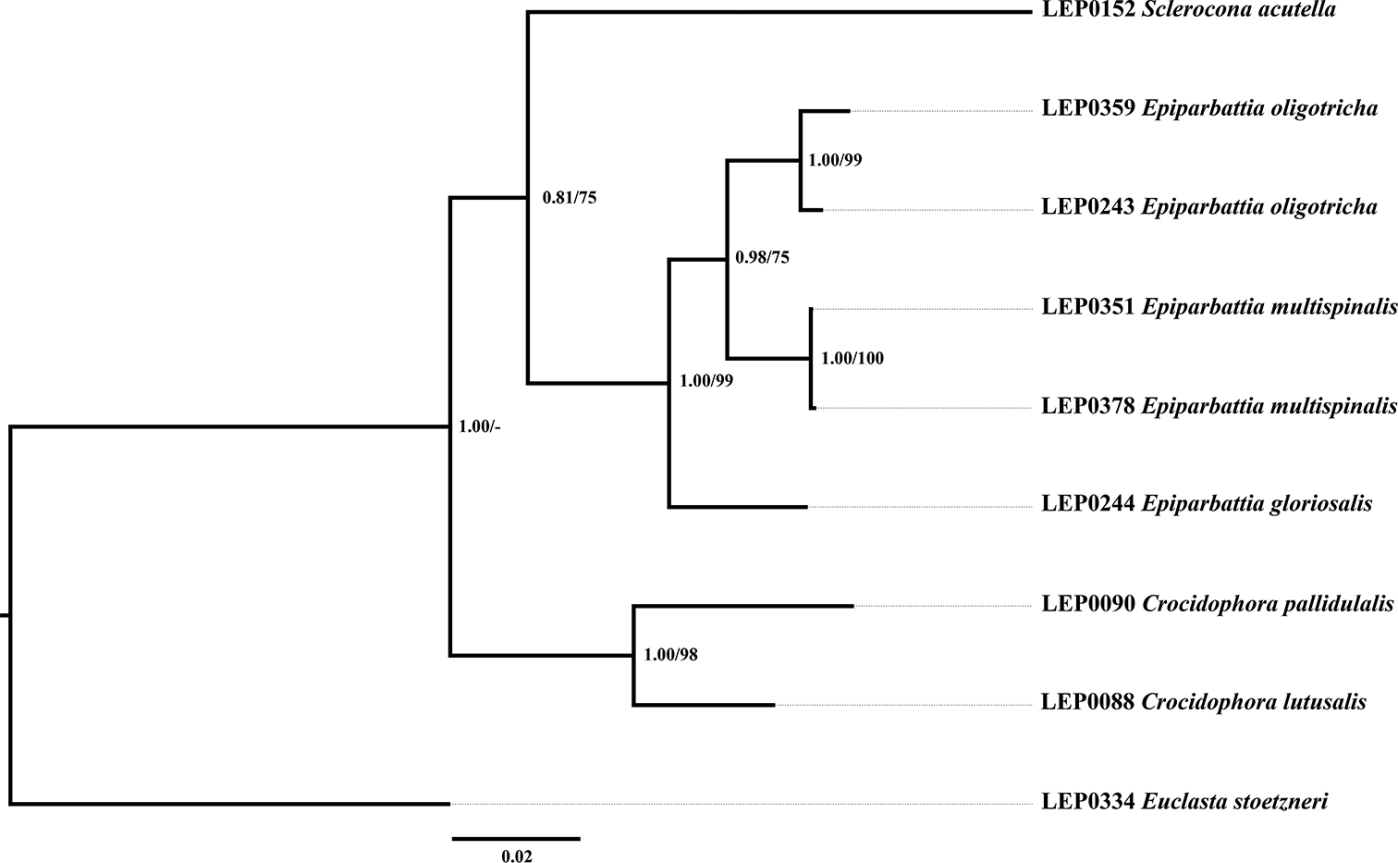
Phylogenetic hypothesis inferred from Bayesian inference. Numbers on branches indicate Bayesian posterior probabilities and ML bootstrap values respectively.

The concatenated dataset of four genes consisted of 2510 nucleotide positions (658 for *COI*, 468 for *16S rRNA*, 619 for *28S rRNA*, and 765 for *EF-1*α). Both BI and ML analyses of the concatenated dataset inferred congruent topologies with only subtle differences in posterior probability and bootstrap values (Fig. 11). The monophyly of *Epiparbattia* is robustly supported (PP = 1.00, BS = 99), the genus *Sclerocona* and *Epiparbattia* are sister groups with moderate support. The results of the current phylogenetic analyses support the placement of *E. multispinalis* sp. nov. in *Epiparbattia*, with *E. oligotricha* as its sister species, with strong support in the BI analysis (PP = 0.98, BS = 75).

Pairwise distances of the barcoding region (*COI*) are given in Table 2. The genetic distances between *Epiparbattia* and other genera range from 8.5% (*Crocidophora*) to 15.5% (*Euclasta*). Interspecific genetic distances within *Epiparbattia* range from 5.6% (*E. oligotricha* to *E. multispinalis*) to 7.9% (*E. oligotricha* to *E. gloriosalis*), while intraspecific genetic distances range from 0.2% (*E. multispinalis*) to 1.9% (*E. oligotricha*).

**Table 2.** Pairwise distances of the *COI* barcode region based on Kimura-2-parameter model (intraspecific distances are highlighted in bold).

		1	2	3	4	5	6	7	8
**1**	SYSU-LEP0334 *Euclasta stoetzneri*								
**2**	SYSU-LEP0088 *Crocidophora lutusalis*	0.133							
**3**	SYSU-LEP0090 *Crocidophora pallidulali*s	0.136	0.077						
**4**	SYSU-LEP0243 *Epiparbattia oligotricha*	0.138	0.098	0.085					
**5**	SYSU-LEP0359 *Epiparbattia oligotricha*	0.133	0.101	0.089	**0.019**				
**6**	SYSU-LEP0351 *Epiparbattia multispinalis*	0.153	0.103	0.098	0.056	0.070			
**7**	SYSU-LEP0378 *Epiparbattia multispinalis*	0.155	0.105	0.099	0.058	0.071	**0.002**		
**8**	SYSU-LEP0244 *Epiparbattia gloriosalis*	0.135	0.098	0.089	0.072	0.079	0.067	0.069	
**9**	SYSU-LEP0152 *Sclerocona acutella*	0.118	0.113	0.092	0.092	0.103	0.087	0.089	0.096

## Discussion

The monophyly of *Epiparbattia* is robustly supported by the results of the molecular analysis. Three species can be recognized as members of *Epiparbattia* by a series of external and genital characters provided above in the diagnosis of the genus. As is apparent from the tree topology (Fig. 11), *E. multispinalis* is more closely related to *E. oligotricha* than to *E. gloriosalis* which makes good sense with respect to the similar hair-like editum in male genitalia (Figs 6, 7). According to the tree topology (Fig. 11), *Epiparbattia* is more colsely related to *Sclerocona*. Species of *Epiparbattia* and *Sclerocona* have two foveae on the forewing in males, a developed and sclerotized lamella postvaginalis, and a weakly developed, almost reduced antrum in females.

Additionally, several pyraustine genera, *Anamalaia* Munroe & Mutuura, 1969, *Lepidoplaga* Warren, 1895, *Limbobotys* Munroe & Mutuura, 1970 and *Torulisquama* Zhang & Li, 2010, are similar to *Epiparbattia* and *Sclerocona* by bearing fovea (at least one fovea) on the forewing, minute basal and apical outer spurs of hindleg in males, as well as a developed and sclerotized lamella postvaginalis in females, but can still be distinguished from each other by the number and position of the fovea and other genital characters. The relationships among all these genera need to be further studied.

## Supplementary Material

XML Treatment for Epiparbattia

XML Treatment for Epiparbattia gloriosalis

XML Treatment for Epiparbattia oligotricha

XML Treatment for Epiparbattia multispinalis
